# Supplementary material for: Hydrolysis of palm kernel meal fibre using a newly isolated Bacillus subtilis F6 with high mannanase activity
Source: Bioresour Bioprocess. 2024 Dec 25;11(1):113. doi: 10.1186/s40643-024-00826-9 (PMC11669640; doi:10.1186/s40643-024-00826-9)
Supplement: Supplementary file 1 — Additional file 1. [file 40643_2024_826_MOESM1_ESM.docx]

**Supplementary figure and table:**


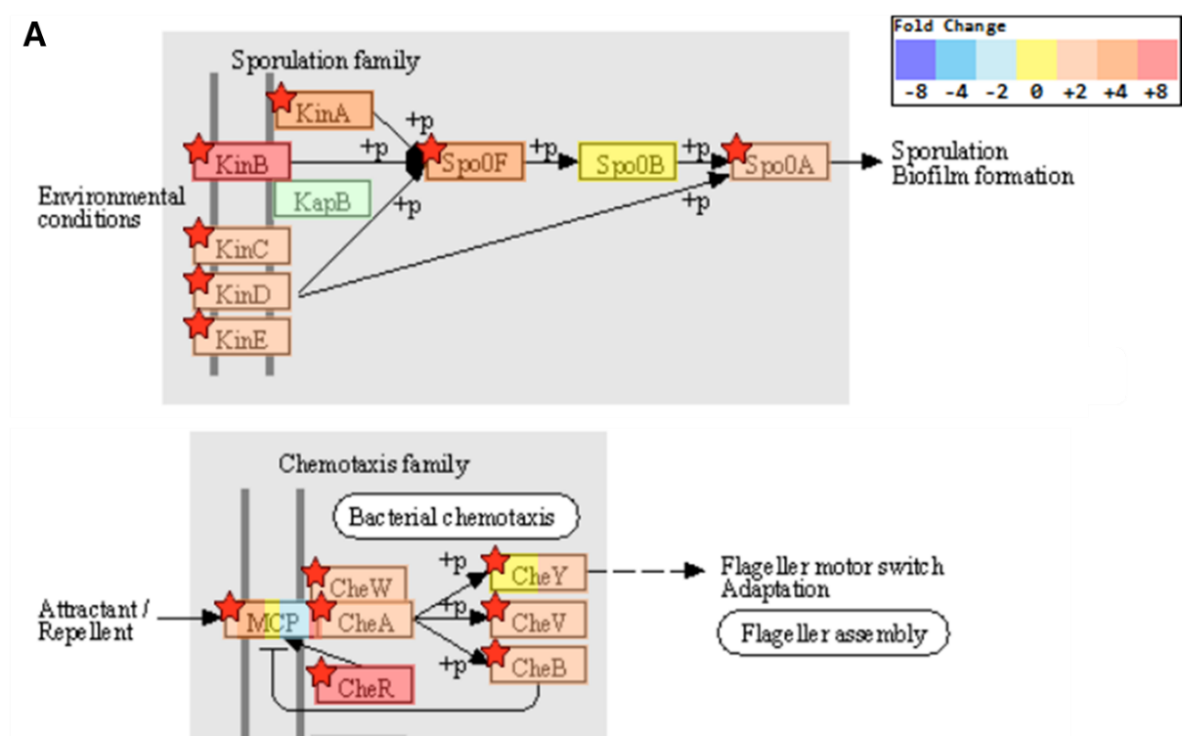


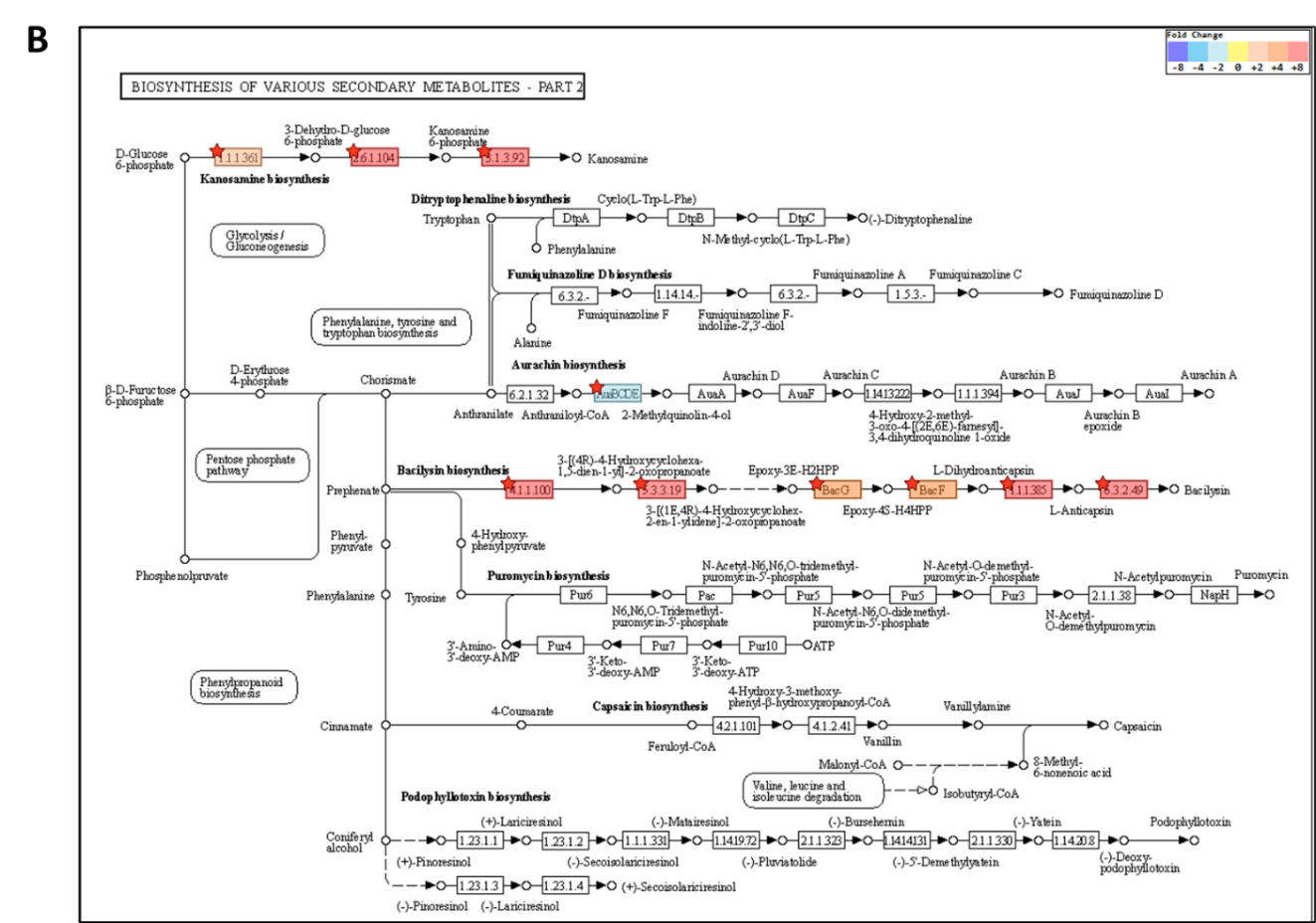


**Figure S1** **Upregulated expression of genes involved in other pathways** **in *B. subtilis* F6 after 6 h-solid-state fermentation of PKM.** Upregulation of gene expression was observed in the process of (A) sporulation, biofilm formation, flagellar assembly and (B) kanosamine and bacilysin biosynthesis. Significant pathway module is marked with red star. Fold change values of DEG are shown in colours. The fold change is the expression level after SSF with respect to before SSF. White box of pathway map is module that is not relevant to the species.

**Table S1** **LC-MS analysis of soluble proteins in PKM fermented by *B. subtilis* F6**

| Protein Group | Protein ID | Accession | -10lgP | Coverage (%) | #Peptides | #Unique | PTM | Avg. Mass | Description |
| --- | --- | --- | --- | --- | --- | --- | --- | --- | --- |
| 1 | 17740 | ODGIONBF_03226 | 127.63 | 9 | 11 | 11 | N | 55314 | Endoglucanase |
| 6 | 19366 | ODGIONBF_03520 | 85.51 | 15 | 4 | 4 | N | 40890 | Beta-mannosidase |
| 7 | 17743 | ODGIONBF_02870 | 70.57 | 4 | 3 | 3 | N | 56522 | Extracellular neutral metalloprotease |
| 5 | 17746 | ODGIONBF_03122 | 64.45 | 1 | 5 | 5 | Y | 613247 | Polyketide synthase PksN |
| 62 | 21820 | ODGIONBF_02399 | 56.72 | 5 | 1 | 1 | N | 39479 | Peptidase S8 |

Notes: Soluble proteins were extracted from the fermented PKM using water and analysed by LC-MS following trypsin digestion. The *B. subtilis* F6 proteome database was used for protein identification. The table displays the top five proteins with the highest -10lgP scores, indicating a high confidence level in their identification.
